# Multiple and Alternative Sites Make Tau Protein an Adaptable Sticky Surface for the SH3 Domain of Fyn Kinase

**DOI:** 10.1002/anie.202504292

**Published:** 2025-05-23

**Authors:** Roberto Tira, Giulia Leo, Laura Prandini, Carlo Giorgio Barracchia, Mariapina D'Onofrio, Luca Mollica, Stefano Capaldi, Michael Assfalg, Francesca Munari

**Affiliations:** ^1^ Department of Biotechnology University of Verona Verona Italy; ^2^ Department of Medical Biotechnology and Translational Medicine University of Milan Milan Italy

**Keywords:** Molecular recognition, NMR spectroscopy, Protein‐protein interactions, SH3 domain, Tau protein

## Abstract

The interaction between the microtubule associated protein Tau and the tyrosine kinase Fyn is believed to play a pivotal role in the early stage of Alzheimer's disease. Previous studies have identified the SRC Homology 3 (SH3) domain of Fyn as the binding receptor of several proline‐rich motifs in Tau. However, the role of each proline‐rich motif and their interplay in molecular recognition are still unclear. In this work, we investigated the mechanism of Fyn‐SH3 recognition by the multiple PxxP sites inserted within the full‐length Tau protein by using nuclear magnetic resonance (NMR) spectroscopy combined with computational, calorimetric and in‐cell FRET (Förster resonance energy transfer) methods. Both in vitro and in‐cell experiments revealed no single binding site strictly necessary for the binding. Instead, Fyn‐SH3 contacts full‐length Tau on multiple hot spot regions, located over a distance of 85 residues, through global moderate‐to‐low affinity interactions. Beyond two principal regions containing classical PxxP motifs, we identified a novel non‐canonical binding site at the beginning of the microtubule binding domain. Our study indicates that multiple binding sites in Tau are involved in the interaction, making Tau an adaptable recognition surface that can function when single consensus motifs are deleted.

## Graphical abstract

An NMR‐based study, combined with computational, calorimetric and in‐cell FRET methods, sheds light on the complex recognition mechanism between the intrinsically disordered protein Tau and the SH3 domain of Fyn kinase, highlighting how the multiple binding sites present in Tau make the protein an adaptable recognition surface that can function even when single consensus motifs are eliminated.

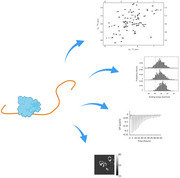

## Introduction

**Figure 1:**
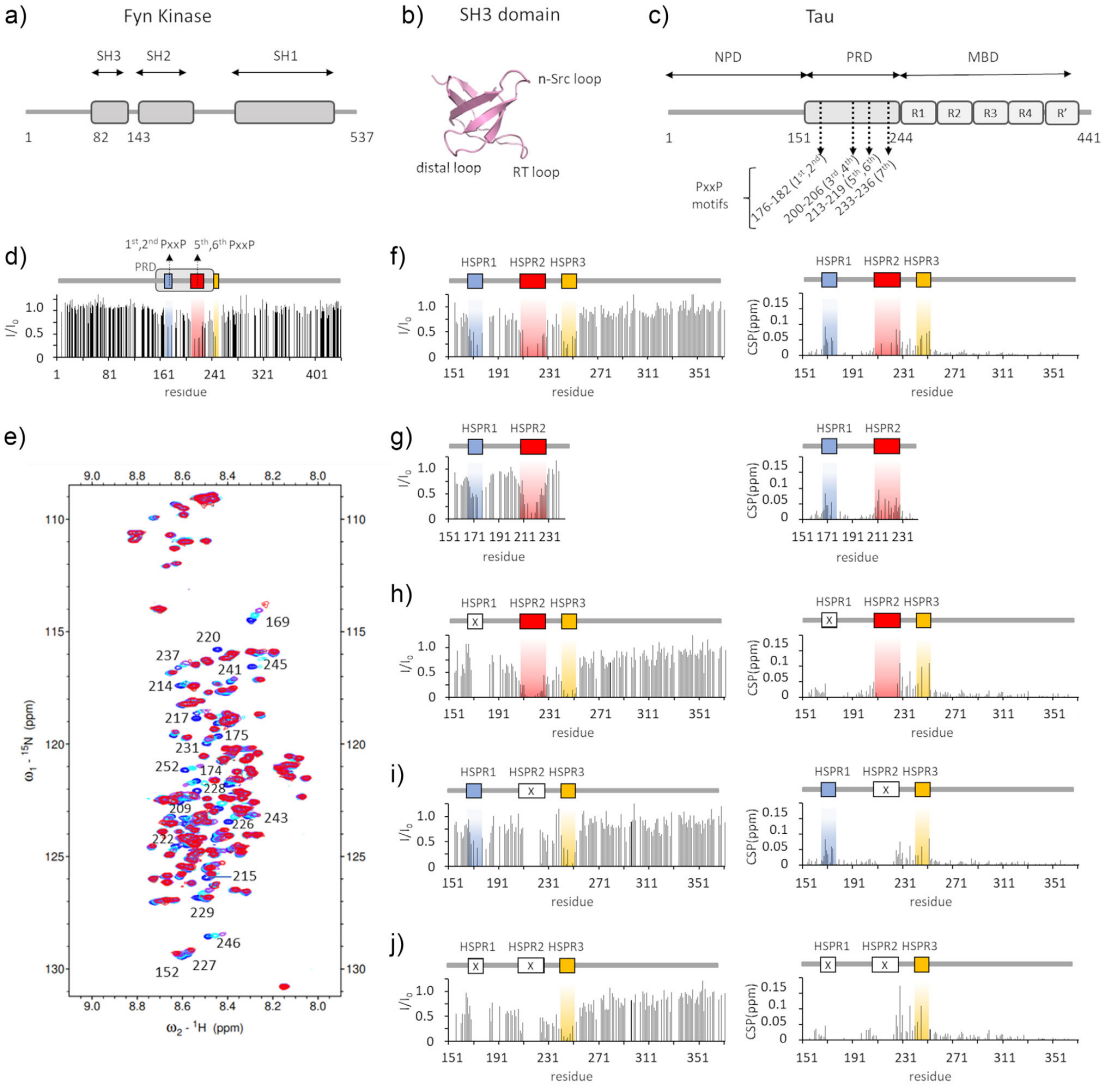
NMR analysis of Tau – Fyn SH3 binding. a) Protein domains of Fyn Kinase. b) Structural model of the Fyn‐SH3 domain based on the 1shf PDB file.^[^ ^38^ ^]^ c) Domains organization of Tau protein (isoform 2N4R). The seven PxxP motifs are indicated as first‐seventh, with the spanning amino acid region. Their sequence is reported in Figure S1A. d‐j) NMR‐binding experiments. Intensity ratio (I/I_0_) of NMR signals from ^1^H,^15 ^N spectra of ^15^N‐Tau constructs 1–441 (d), 151–372 (f), 151–243 (g), 151–372 Δ175–179 (h), 151–372 Δ215–219 (i), 151–372 Δ175–179 Δ215–219 (j) acquired alone (I_0_) and in presence of Fyn‐SH3 (I) at 1:1 molar ratio, versus the Tau sequence. The chemical shift perturbation (CSP) plots are shown on the right. Residues affected by signal overlap were excluded from the analysis. The scheme of the deletion (x) and of the main regions affected (highlighted with coloured boxes), is shown on the top. e) ^1^H,^15 ^N HSQC spectra overlay of ^15^N‐Tau 151–372 alone (blue) and in presence of Fyn‐SH3 at 2:1 (cyan), 1:1 (purple) and 1:2 (red) molar ratio. Labels for selected peaks are shown.

Tau is a microtubule‐associated protein mainly present in the central nervous system (CNS) and localized in neurons axon where it plays a role in assembly of microtubules, contributing to regulation of axonal stability, neurite outgrowth and cargo transport.^[^ ^1^, ^2^ ^]^ Limited amounts of Tau are also physiologically found in other neuronal compartments, the soma and the dendrites at the post‐synapse, where it seems to be implicated in neuronal signaling and synaptic plasticity.^[^ ^3^ ^]^ Tau is best‐known for its major involvement in tauopathies, a vast class of neurodegenerative diseases, which include Alzheimer's disease (AD), characterized by the aberrant hyperphosphorylation and conversion of Tau from a natively unfolded state to amyloid filament structures that accumulate in neurofibrillary tangles (NFTs) and spread throughout the brain.^[^ ^1^, ^2^ ^]^ However, emerging evidence indicates that missorting of Tau from axons to the somatodendritic compartment, induced by various triggers (e.g., Aβ‐oligomers), seems to direct a major neurotoxic activity in the earlier stages of the pathology, resulting in synaptic deficits and neurons damage.^[^ ^4^, ^5^, ^6^ ^]^ These represent early hallmarks of neurodegeneration and are strongly correlated to the cognitive decline observed in AD.^[^ ^3^, ^4^, ^7^ ^]^ A key player of Tau‐mediated synaptic dysfunction is the tyrosine kinases Fyn which, once overly recruited to the post‐synapse through interaction with missorted Tau, enhances the stabilization and activation of the N‐methyl‐D‐aspartate (NMDA) receptors, resulting in neuronal excitotoxicity.^[^ ^4^ ^]^ Additional evidence point to a role of the Fyn‐Tau interaction in neurodegenerative processes: I) Fyn over‐expression was found to exacerbate neuronal deficits in AD models^[^ ^8^ ^]^; II) Fyn can phosphorylate Tau at Y18, a pathological marker of AD filaments^[^ ^9^ ^]^; III) expression of Fyn is altered in AD and Fyn was found to colocalize in the NFTs formed by hyperphosphorylated Tau^[^ ^10^ ^]^; IV) Fyn can drive Tau pathology independently of Aβ^[^ ^11^ ^]^; V) frontotemporal dementia mutant Tau P301L promotes aberrant Fyn nanoclustering in dendritic spines.^[^ ^12^ ^]^ In light of this, the Tau‐Fyn complex is emerging as an appealing drug‐target for the treatment of AD and other tauopathies, and inhibitors of this protein‐protein interaction are already under investigation.^[^ ^13^, ^14^ ^]^ The interaction between Fyn and Tau is mediated by the SRC Homology 3 (SH3) domain of Fyn which is located N‐terminal to the SH2 and the kinase SH1 domains (Figure 1a,b). Typically, SH3 domains recognize hydrophobic PxxP proline‐rich motifs in a left‐handed polyproline type II (PPII) helical conformation through a pair of aromatic grooves. The consensus motif is stronger with XPxXP sequences (wherein X is generally a hydrophobic residue and x is any residue) while the binding in a forward or reverse orientation depends on a positively charged residue flanking the proline rich core.^[^ ^15^, ^16^ ^]^ SH3‐ligand interactions are generally characterized by low affinity and a certain degree of promiscuity. Indeed, binding of partners largely deviating from the consensus motif has also been observed.^[^ ^16^, ^17^, ^18^ ^]^ Interestingly, it has been suggested that SH3 domains contribute to the regulation of Src‐type kinases also through intramolecular interactions with the N‐terminal disordered region typical of these proteins.^[^ ^19^, ^20^ ^]^ Tau protein has a central proline‐rich domain (PRD) containing seven PxxP motifs, which is located between the N‐terminal projection domain (NPD) and the C‐terminal microtubule binding domain (MBD) composed of four repeats R1‐R4 (Figure 1c). The role of these PxxP motifs in Tau‐SH3 interaction has been analyzed in previous studies.^[^ ^21^, ^22^, ^23^, ^24^, ^25^, ^26^, ^27^ ^]^ However, results did not provide a consensus, possibly due to the use of isolated peptides, differently designed mutants and pull‐down methods. Early studies identified the first PxxP motif as the major Fyn‐SH3 binding site in Tau.^[^ ^24^ ^]^ The work of Lau and colleagues highlighted the importance of the fifth/sixth PxxP motifs and reported that individual mutations of the key residues P216 or P219 in Tau reduced SH3‐binding by approximately 35%. However, neither mutation was able to abolish the interaction, suggesting that additional regions significantly contribute to binding.^[^ ^26^ ^]^ Another study corroborated the importance of the fifth/sixth PxxP motifs over the seventh PxxP motif, and the fact that the Fyn‐Tau interaction persists, albeit with a significant reduction, when P216 is mutated to alanine.^[^ ^25^ ^]^ However, the contribution of the first, second and third motif was not investigated in that work. It seems therefore clear that the binding between Tau and the SH3 domain of Fyn involves multiple regions of Tau, and that the complexity of the system requires the analysis of the full Tau protein, since the use of isolated motifs has led to conflicting or incomplete results.

In this work, we employed nuclear magnetic resonance (NMR) spectroscopy in combination with computational, calorimetry and in cells‐FRET (Förster resonance energy transfer) experiments to untangle the complexity of Fyn‐SH3 recognition by the multiple PxxP sites present in the Tau protein. Our structural and quantitative analysis revealed no single binding site in Tau strictly necessary for the binding. Instead, multiple hot spot regions, located on a stretch of 85 residues, are engaged in a global moderate‐to‐low affinity interaction. Remarkably, beyond two main hot spot regions containing classical PxxP motifs within residues 176–182 and 213–219, we identified an unexpected binding site within residues 241–253 that involves the beginning of the microtubule binding domain. This novel site characterized by alternating proline residues binds, although with lower affinity, to the same surface used by SH3 to recognize the PxxP motifs. Our data highlight the versatility of Tau in interacting with Fyn‐SH3 through very different binding regions: this ability could find biological significance in sustaining the association between the two proteins even when specific consensus motifs are compromised, for example as a consequence of post‐translational modifications.

## Results and Discussion

### The SH3 Domain of Fyn Contacts Full‐length Tau at Multiple Sites

In the longest isoform of Tau expressed in the adult human CNS (isoform 2N4R), the proline‐rich domain spans residues 151–244 and contains 22 proline residues, some of which form seven PxxP motifs: each of the regions 176–182, 200–206 and 213–219 contains two partially overlapped motifs, while region 233–236 contains a single PxxP motif (Figure 1c and Figure S1a). Among these, only the sequence ^215^LPTPP^219^ respects the strong consensus motif XPxXP.

To understand the individual contribution of the proline‐rich motifs in contacting the Fyn‐SH3 domain in the context of the full‐length Tau protein, the binding of SH3 to ^15^N‐labeled Tau isoform 2N4R was monitored by NMR spectroscopy. NMR is particularly suited for the study of protein‐protein interactions involving multiple sites, as it allows for the analysis of distinct binding interfaces at atomic‐level in a single experiment. Notably, NMR has proven to be an excellent method for the structural characterization of multi‐site transient protein‐Tau complexes.^[^ ^28^, ^29^, ^30^, ^31^, ^32^ ^]^ In the 2D ^1^H‐^15 ^N correlation spectra (^1^H‐^15^N‐HSQC or ^1^H‐^15^N‐HMQC spectra) of proteins, each peak derives from a ^1^H‐^15 ^N correlation and is assigned to the backbone amide group of each amino acid residue, except for prolines.

The addition of unlabeled Fyn‐SH3 to ^15^N‐Tau resulted in shifts and intensity reduction for a selection of ^1^H‐^15 ^N NMR signals (Figure S1B), indicating the presence of specific interactions between the two proteins. The signal loss (I/I_0_) reported in Figure 1d shows that the main perturbed region is between residues 170 and 253, within the proline‐rich domain to the beginning of the R1 repeat. Since the 1–150 region and the very C‐terminal part of Tau were not significantly perturbed, we next used a shorter construct restricted to the 151–372 region, to reduce signal overlaps and to allow more accurate analysis of the chemical shift perturbation (CSP). A superimposition of NMR‐binding profiles of Tau2N4R and of Tau151‐372 upon binding to SH3 (Figure S1C) shows that the same pattern of reduction in signals intensity was obtained. Based on the loss of signal intensity, we identified three main regions that are highly perturbed (with I/I_0_ <0.55) upon SH3‐Tau binding: between residues 167 and 178, between residues 207 and 229, and between residues 241 and 253 (Figure 1f). These regions, hereafter referred to as hotspot regions 1, 2 and 3 (HSPR1, 2 and 3), were also clearly visible in the experiment with the full‐length protein (Figure 1d and Figure S1C). Residues within regions 168–175, 222–229, 235–237 and 241–252, which are within or close to the identified HSPRs, show significant shifts in peak position, and signals of residues 215, 217 and 220 in HSPR2 disappeared upon complex formation (Figure 1e,f). On the other hand, peaks of residues within region 199‐SPGSPGTP‐206 remained almost unperturbed (Figure 1f), suggesting that the two PxxP motifs there included do not bind SH3 in the context of a complete proline‐rich domain. This was confirmed by testing the binding of SH3 to a shorter construct lacking the HSPR3 and the MBD (Tau 151–243): this showed signals perturbation within HSPR1 and HSPR2, while the region 199–207 remained unperturbed (Figure 1g and Figure S1D) as in the longer protein. Also, the 234–239 region appeared unaffected, questioning the direct involvement of the seventh PxxP motif in binding to Fyn‐SH3 when HSPR1 and HSPR2 are present. However, in the longer Tau151‐372 construct, the shifts observed for residues 235–237 could reflect interactions of the 233–236 region (which can be referred as HSPR3‘) that are secondary to the binding of SH3 to HSPR3.

Overall, we identified three hot spot regions in full‐length Tau that are primarily involved in interaction with the SH3 domain of Fyn: two of them, HSPR1 and HSPR2, contain the PxxP motifs within ^175^TPPAP^179^, ^178^APKTP^182^, ^212^TPSLP^216^ and ^215^LPTPP^219^. Conversely the third, HSPR3, located in the very first portion of the R1 repeat of the MBD, lacks the typical SH3 binding motif. However, it contains many hydrophobic residues and prolines (as in ^247^PVPMP^251^), which could provide an alternative binding site for SH3, as already reported for other atypical SH3 binding modes.^[^ ^18^ ^]^

To evaluate the relative influence of one binding region on the others, we used Tau deletion mutants to remove the PxxP motifs in HSPR1 (Tau151‐372 Δ175–179), in HSPR2 (Tau151‐372 Δ215–219) or in both (Tau151‐372 Δ175–179, Δ215–219).

NMR experiments on ^15^N‐Tau Δ175–179 demonstrated that the key motifs in HSPR1 are not essential for the binding of SH3 to Tau: signal perturbation persists for both HSPR2 and HSPR3, with the latter experiencing a larger attenuation of signals intensity (Figure 1h) with respect to the unmutated sequence. Also, the signal intensity of residues before Gln165 appears more affected, which can be attributed to increased non‐specific contacts in this region.

The SH3‐Tau interaction occurs despite deletion of PxxP motifs within HSPR2 as well: in fact, signals perturbations of ^15^N‐Tau Δ215–219 are visible for both HSPR1 and HSPR3 (Figure 1i). We also noted that shifts of peaks in the 226–229 region and of residue 237 persist, indicating that their perturbation is independent of HSPR2.

The perturbation of NMR signals in HSPR3 is clearly visible after deletion of both the 175–179 and 215–219 regions (Figure 1j), thus strengthening the hypothesis that HSPR3 is an authentic binding site for SH3. Significant loss of signal is also observed for many residues N‐terminal to HSPR3 and shifts of peaks in the 226–229 region and of residues 231 and 237 are larger, probably due to increased contacts at these sites upon the deletion of the PxxP motifs in HSPR1 and HSPR2.

NMR‐based competition experiments demonstrated that a molar excess of isolated Tau peptide 170–184 could compete with the binding of SH3 to the HSPR3 site on the Tau 151–372 Δ175–179 Δ215–219 mutant. This effect is visible as a recovery of NMR signal intensity for residues in HSPR3, and small CSPs (Figure S1E). A similar result was obtained using an excess of Tau peptide 210–224 (Figure S1F). These experiments thus confirmed that HSPR3 is an authentic binding site for SH3, and that the HSPR1‐2‐3 regions constitute alternative sites for the interaction with the PxxP‐binding groove of Fyn‐SH3 domain.

### Fyn‐SH3 Binds Different Regions of Tau Through the Same Site

**Figure 2:**
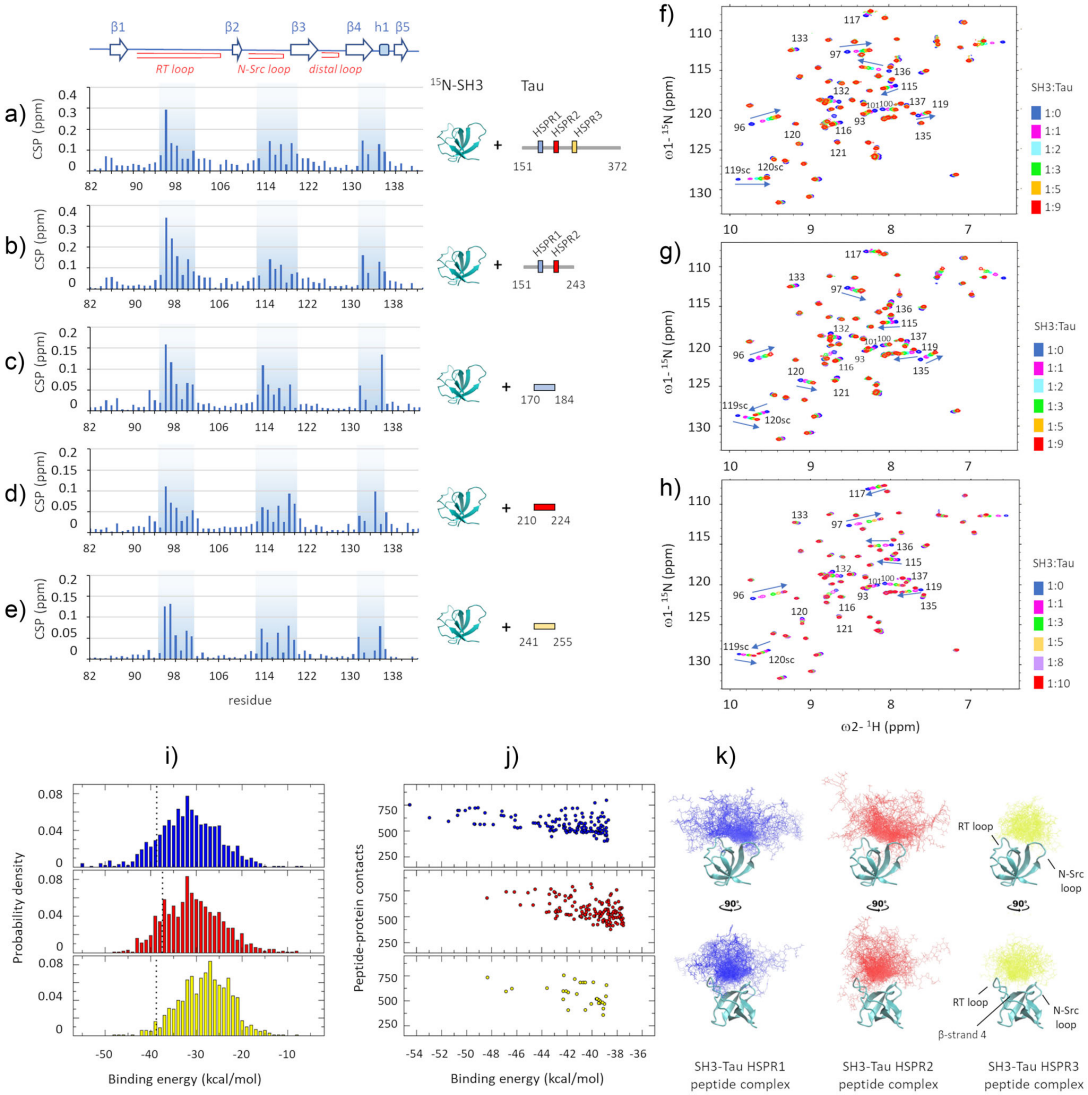
NMR and computational analysis of Tau peptides binding to Fyn‐SH3. CSP‐plots of ^15^N‐labeled SH3 in the presence of Tau fragments 151–372 (a), 151–243 (b) and of Tau peptides 170–184 (c), 210–224 (d) and 241–255 (e) at 1:1 molar ratio. Overlay of ^1^H,^15^N‐HMQC spectra of Fyn‐SH3 in the presence of Tau peptide 170–184 (f), Tau peptide 210–224 (g) or Tau peptide 241–255 (h) at different molar ratios (indicated on the right). The assignment of selected resonances is reported for comparison. j‐k: Computational structural characterization of the Fyn SH3‐Tau HSPR peptide complexes (blue: HSPR1; red: HSPR2; yellow: HSPR3) by SMD and molecular docking. i) Distribution of binding energies resulting from molecular docking. Dotted vertical lines indicate the threshold set at one standard deviation below the mean binding energy, calculated from 1000 samples. j) Maps of peptide‐protein contacts versus binding energy. k) Ensembles of representative conformers for the SH3‐Tau HSPR1 (144 structures), SH3‐Tau HSPR2 (144 structures) and SH3‐Tau HSPR3 (31 structures) peptide complexes, selected from the respective lowest energy portion of the binding energy distribution.

Having identified the SH3‐binding interface on Tau, we sought to evaluate the interaction surface for Tau constructs of various lengths on ^15^N‐SH3 by ^1^H,^15^N‐HMQC experiments. The addition of unlabeled Tau fragments 151–372 or 151–243, both containing the proline rich domain, resulted in a similar perturbation profile, mainly affecting the same region of the protein which includes residues within the RT loop, the N‐Src loop, the β‐strand 4 and the small helix 1 (Figures 2a,b and Figure S2): major CSPs were observed for residues within regions 95–101, 113–120 and 132–136, in line with the CSP pattern observed for other Fyn‐SH3 interactions.^[^ ^17^ ^]^

To evaluate whether Fyn utilizes the same residues to recognize the different proline rich motifs of Tau, we tested binding with three synthetic peptides of 15 amino acids with sequence centered on the previously identified recognition motifs: Tau170‐184, Tau210‐224 and Tau241‐255 (named HSPR1,2,3 peptides). Peptide‐SH3 titration experiments followed by ^1^H,^15^N‐HMQC spectra and the corresponding CSP analysis (Figure 2c–h) highlighted how the three Tau HSPR peptides form fast‐exchange complexes and perturb peaks of residues belonging to the same SH3 regions affected by the longer constructs of Tau. However, a different behavior has been observed for selected residues, suggesting their diverse involvement in Tau recognition: among the aromatics that typically form the PxxP‐binding groove, while the peaks of residues Y137 and Y93 change little, the amide resonance of W119 shows a large shift and with opposite directions in the SH3‐HSPR1 complex compared to the SH3‐HSPR2/3 complexes. Outside the PxxP‐binding groove, a large shift of the W120 resonance was observed with Tau210‐224 and Tau241‐255, but not with Tau170‐184, indicating that this aromatic residue is not involved in the recognition of the latter peptide. Furthermore, a large shift of the N136 resonance was observed with Tau170‐184 and Tau241‐255, but not with Tau210‐224, while a large shift of S135 resonance was observed only with Tau210‐224 (Figure 2f–h).

The obtained NMR data were used to build structural models of the SH3‐Tau HSPR peptide complexes. After generating an ensemble of structures of each of the 15‐residues Tau peptide by scaled molecular dynamics (SMD), we performed rigid body docking using peptides conformers corresponding to the centers of the trajectory representative clusters and the X‐ray crystal structure of Fyn‐SH3. Structural models of Tau peptide‐SH3 complexes were generated based on our CSP data in the form of ambiguous restraints (see supplementary methods).

The analysis of the lowest‐energy structures (i.e., the ones belonging to the left tail of the overall binding energy distribution, Figure 2i) evidenced that all the HSPR Tau conformers lean on the same region of the SH3 surface formed by residues of the RT loop, the N‐Src loop, the β‐strand 4 and the small helix 1 (Figure 2k). Among the three SH3‐HSPR peptide complexes, that with the Tau170‐184 peptide presents the lowest binding energy and the largest number of contacts (Figure 2i,j). The two parameters progressively decrease in the SH3‐HSPR2 and SH3‐HSPR3 complexes, suggesting a descending ranking of binding affinity with SH3‐HSPR1 > SH3‐HSPR2 > SH3‐HSPR3.

### Multiple SH3‐binding Sites Compensate for the Absence of Single PxxP Motifs

**Figure 3:**
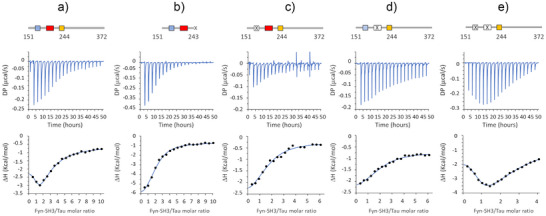
ITC analysis of Fyn‐SH3 binding to Tau. ITC measurements of Fyn‐SH3 titrated into Tau fragments 151–372 (a), 151–243 (b), 151–372 Δ175–179 (c), 151–372 Δ215–219 (d), 151–372 Δ175–179 Δ215–219 (e). The upper and lower panels show the raw thermograms (DP, differential power), and the integrated heats corrected for the heat of dilution (ΔH), respectively. The blue line in the latter panels represents the fit curve.

To estimate the relative strength of the hot spot regions identified on Tau, we measured the binding affinities for Fyn‐SH3 evaluating each site when isolated as single peptide or in combination within a longer construct. Binding isotherms for each of the three synthetic peptides Tau170‐184, Tau210‐224 and Tau241‐255 were derived from HMQC‐based titrations on ^15^N‐labeled SH3: the fitted Kd values (127, 163 and 327 µM, respectively, see Figure S3) are in line with the ranking of binding affinities SH3‐HSPR1 > SH3‐HSPR2 > SH3‐HSPR3, indicated by computational methods. The lower affinity for the HSPR3 peptide is justified by the lack of two properly spaced proline residues which form the typical PxxP motif recognised by the SH3 proline‐binding groove. The NMR‐derived Kd values highlight how isolated peptides individually bind SH3 with very low affinity: this suggests that the proline‐rich motifs must be located in a longer protein region to ensure additional interactions and higher binding energy. Indeed, isothermal titration calorimetry (ITC) performed on longer Tau constructs evidenced that binding affinity increases with the length of the protein (Figure 3 and Table 1). Remarkably, the isotherm resulting from titrating SH3 into Tau151‐372, which contains all the three HSPRs, exhibits a U‐shape at the beginning of the titration. The U‐shape isotherm in ITC data, where initial integrated heats move to increasing negative enthalpy, while later heats tend to decreasing negative enthalpy, indicates the occurrence of distinct binding events: it hints that interaction between the protein and its ligand occurs at more than one site, with the binding sites having clearly distinguishable thermodynamic features.^[^ ^33^, ^34^ ^]^ This biphasic profile was better fit with a two‐sets of site binding model. Although absolute values should be taken with caution because of the complexity of the system and the inherent fitting uncertainty within this range of weak affinities, ITC data indicate that Tau151‐372 contains multiple binding sites for Fyn‐SH3, with moderate‐low affinity in the micromolar range (N_I_∼1, Kd_I _= 2.5 µM; N_II_∼3, Kd_II _= 38 µM) (Figure 3a and Table 1). On the other hand, binding of SH3 to the Δ175–179 or Δ215–219 deletion Tau mutants or to the Tau 151–243 construct (all three lacking a single HSPR site) generated uniphasic isotherms featuring as single transition, probably because contributed by two sites with overlapping thermodynamic features (Figure 3b–d). Thus, these simplest curves were better fit with the one‐set of site‐binding model. The obtained results indicate that disabling one HSPR moderately modifies the binding affinity: Tau Δ175–179, lacking the PxxP motifs in HSPR1, binds to SH3 with N = 2 and Kd of 12 µM; Tau Δ215–219, lacking the PxxP motifs in HSPR2, binds to SH3 with N = 2 and Kd of 13 µM, and Tau 151–243, lacking the HSPR3, binds to SH3 with N = 2 and Kd of 11 µM (Figure 3b–d and Table 1).

Interestingly, the isotherm resulting from the titration of SH3 into Tau Δ175–179 Δ215–219 exhibits a pronounced biphasic profile (Figure 3e). As in the case of Tau151‐372, the biphasic curve was better fit with a two‐sets of sites binding model, which provided two sets of parameters: I) N_I_∼1, Kd_I _= 10 µM and II) N_II_∼5, Kd_II _= 347 µM (Table 1).

**Table 1:** ITC‐derived thermodynamic parameters of Tau – Fyn SH3 interaction obtained with the MicroCal PEAQ‐ITC Analysis Software. The fitted values for dissociation constant Kd, change in enthalpy ΔH, and number of sites N, are reported, together with the values of ΔG (change in Gibbs free energy) and ‐TΔS (where T is temperature and ΔS is the change in entropy). The given errors come from data fitting (Kd, ΔH, N) or from error propagation (ΔG, –TΔS).

Tau fragment	Kd (µM)	ΔH (kcal/mol)	ΔG (kcal/mol)	–TΔS (kcal/mol)	N (sites)
Tau 151–372	Kd_I_ = 2.5 ± 1.1 Kd_II_ = 37.6 ± 22.7	ΔH_I_ = −1.5 ± 0.9 ΔH_II_ = −3.2 ± 0.1	ΔG_I_ = −7.6 ± 0.3 ΔG_II_ = −6.0 ± 0.4	‐TΔS_I_ = −6.1 ± 1.2 ‐TΔS_II_ = −2.9 ± 0.5	N_I_ = 0.8 ± 0.4 N_II_ = 3.4 ± 0.3
Tau 151–243	Kd = 11.0 ± 2.5	ΔH = −6.6 ± 0.6	ΔG = −6.8 ± 0.1	‐TΔS = −0.2 ± 0.7	N = 2.0 ± 0.1
Tau 151–372 Δ175‐179	Kd = 12.2 ± 5.4	ΔH = −2.7 ± 0.5	ΔG = −6.7 ± 0.3	‐TΔS = −4.0 ± 0.8	N = 1.7 ± 0.1
Tau 151–372 Δ215‐219	Kd = 12.7 ± 4.4	ΔH = −1.9 ± 0.3	ΔG = −6.7 ± 0.2	‐TΔS = −4.8 ± 0.5	N = 2.0 ± 0.1
Tau 151–372 Δ175‐179 Δ215‐219	Kd_I_ = 10.3 ± 2.2 Kd_II_ = 347 ± 79	ΔH_I_ = −1.9 ± 0.2 ΔH_II_ = −4.9 ± 0.1	ΔG_I_ = −6.8 ± 0.1 ΔG_II_ = −4.7 ± 0.1	‐TΔS_I_ = −5.0 ± 0.3 ‐TΔS_II_ = +0.1 ± 0.2	N_I_ = 0.7 ± 0.03 N_II_ = 5.4 ± 0.4

Thus, these data evidence that the various Tau constructs are all able to bind SH3 in a moderate‐to‐low affinity range, and that the deletion of a PxxP site can be buffered by the remaining interacting regions of Tau.

### Tau‐Fyn Intracellular Interaction Occurs Despite Deletion of Single PxxP Motifs

**Figure 4:**
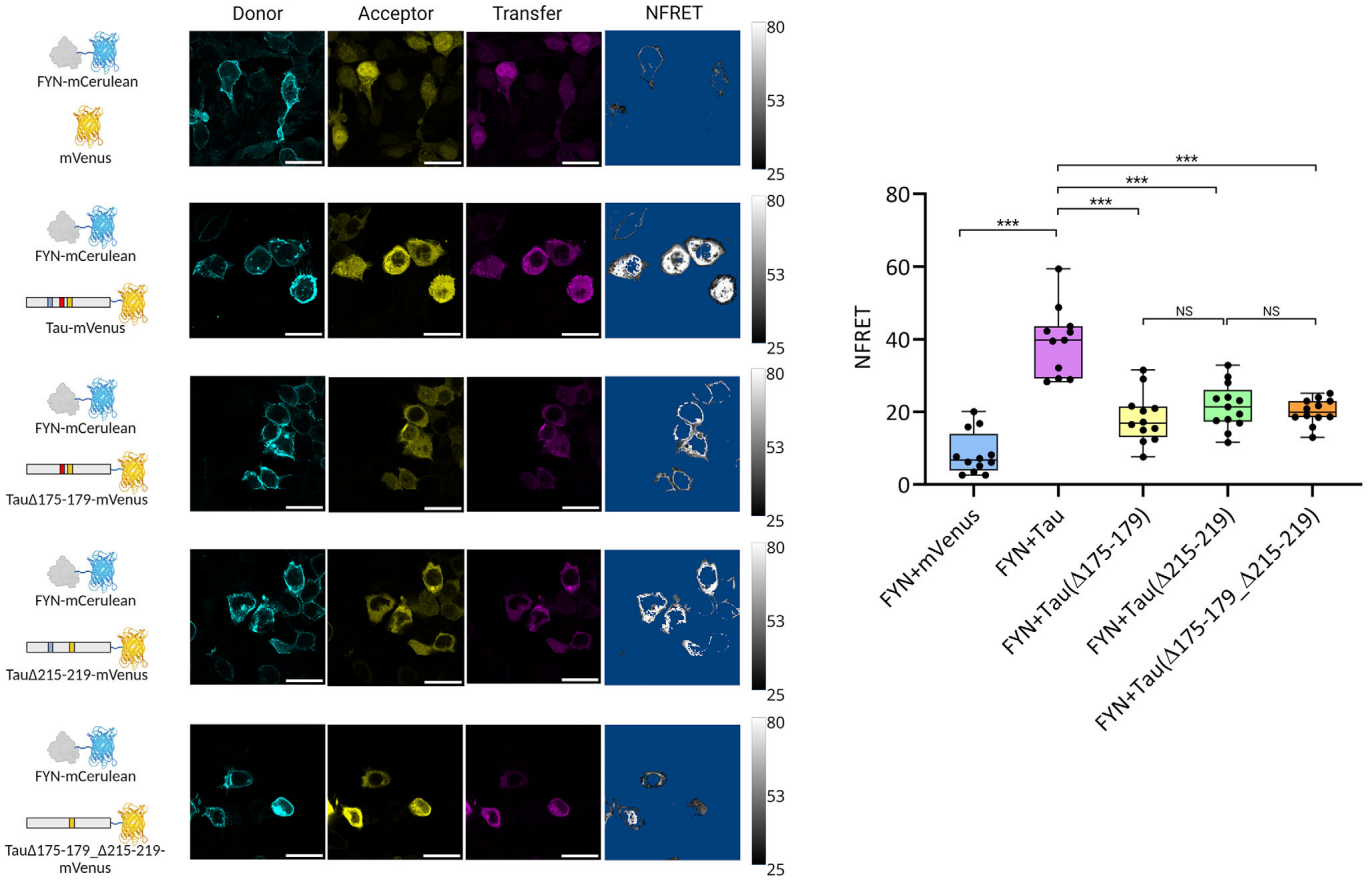
Representative images of the three fluorescence channels (donor, acceptor and transfer channel) and the normalized FRET (NFRET) for different combinations of FRET pairs (indicated on the left). The scale bars are 25 µm. The NFRET images are shown with the same greyscale level. Box‐plots showing the distribution of the average NFRET intensity in individual cells for the same combination of FRET pairs are reported on the right. Experimental values are shown as black dots (*** *p* < 0.005, NS: not significant (one‐way ANOVA with Tukey HSD post‐hoc test; *N* = 12, 11, 12, 13 and 12).

Having defined the relative contribution of different protein regions in the formation of the Tau‐Fyn SH3 complex through biophysical analysis, we reasoned to validate our recognition model using the entire Fyn and Tau proteins in cells. We thus generated a FRET‐based biosensor in Hek293T cell line, where Fyn was fused to a C‐terminal mammalian *Cerulean fluorescent protein* to generate the FRET donor (Fyn‐mCerulean), while Tau (or its deletion mutants) was fused to a C‐terminal mammalian *Venus fluorescent protein* to generate the FRET acceptor (Tau‐mVenus). Cells were transiently transfected with a plasmid that allows the simultaneous co‐expression of both fluorescent proteins, and their expression and interaction were visualised by analysis of a series of confocal images (see supporting information for a detailed description of the acquisition and analysis protocols). The measured normalised FRET (NFRET) values for individual cells, reported in the box‐plot of Figure 4, were used to quantify the Fyn‐Tau interaction. The difference in NFRET values between HEK293T cells co‐expressing Fyn‐mCerulean and Tau‐mVenus (NFRET = 39.5 ± 9.5), with respect cells co‐expressing Fyn‐mCerulean and isolated mVenus (NFRET = 8.5 ± 5.8), validated the robustness of the method in capturing the interaction between Fyn and Tau in our biosensor cellular model. Of notice, NFRET appears more evident in the cytosolic fraction, although Fyn was found distributed in both the plasma membrane and cytoplasm (Figure 4). Inactivation of the PxxP motifs in HSPR1 through deletion of the 175–179 sequence resulted in halving of the NFRET value (NFRET = 18.3 ± 6.9), in line with the importance of the first/second PxxP motifs in mediating the SH3‐Tau interaction. Inactivation of the PxxP motifs in HSPR2 through the Δ215–219 mutant had a comparable effect in decreasing the NFRET value and the interaction (NFRET = 21.6 ± 6.2). Interestingly, the double Tau Δ175–179 Δ215–219 mutant displayed average NFRET value (NFRET = 20.1 ± 3.6) similarly to the Tau Δ175–179 and Tau Δ215–219 mutants (Figure 4), suggesting that the intracellular interaction between the two full‐length proteins does not decrease linearly with the removal of multiple PxxP motifs.

Overall, these experiments indicate that although the first/second and fifth/sixth PxxP motifs in HSPR1 and HSPR2 are important in mediating the binding between the two proteins in cells, other regions of Tau provide a basal level of interaction.

## Discussion

Compelling evidence indicates that the Tau‐Fyn interaction might be crucial in the synaptic damage observed at early stages of neurodegeneration in AD.^[^ ^3^, ^4^, ^5^, ^6^ ^]^ In this regard, high‐resolution information of this molecular recognition event could improve our understanding of the key mechanisms that regulate proteins missorting to the post‐synapse and provide a better framework for the design of selective inhibitors targeting synaptotoxicity in tauopathies. In this work, we have investigated the binding of full‐length Tau with Fyn‐SH3 using NMR spectroscopy, the most powerful high‐resolution technique for the *in‐solution* study of transient protein‐protein interactions involving multiple sites. Three main regions of interaction in Tau were identified: i) 167–178, ii) 207–229 and iii) 241–253 (Figure 1), which overall provide a moderate‐to‐low binding affinity for SH3 (Figure 3 and Table 1). Our analysis evidenced that the first and second HSPRs, which include the first/second PxxP motifs and the fifth/sixth PxxP motifs respectively, mainly contribute to the binding (Figures 2 and S3). However, none of these PxxP motifs seems strictly necessary, since their deletion did not abolish the Tau‐Fyn‐SH3 interaction, as observed in NMR, ITC and in cell‐FRET experiments (Figures 1, 3 and 4 and Table 1). On the other hand, the third‐fourth and seventh PxxP motifs appear to be less or not involved in binding, in agreement with previous works,^[^ ^21^, ^35^ ^]^ and a possible explanation could be that amino acid residues upstream and downstream of these motifs contribute unfavourably to their binding. Interestingly, although the HSPR3 lacks a classical PxxP motif, it constitutes a genuine binding site for SH3: the HSPR3 peptide could in fact bind, albeit with lower affinity, to the same region of SH3 that is recognised by the HSPR1 and HSPR2 peptides (Figures 2 and S3). NMR‐based competition experiments performed with an excess of isolated HSPR1 or HSPR2 peptide corroborated the authenticity of HSPR3 as an alternative ligand of the PxxP‐binding groove in Fyn‐SH3 (Figure S1). Our NMR data also showed that HSPR3 appears to be primarily involved in binding when HSPR1 and HSPR2 are deleted (Figure 1). In this case, a significant perturbation of NMR signals upon Tau binding to SH3 were also observed for several residues located upstream of HSPR3, such as the region 226–229 and residues 231 and 237 (Figure 1), which could reflect the increase of additional binding events occurring upon deletion of the principal PxxP motifs in HSPR1 and HSPR2.

Additional insights on the mechanism of Tau‐SH3 recognition can be obtained by analysis of the thermodynamic data reported in Table 1. Interestingly, the Tau 151–243, Tau 151–372 Δ175–179 and Tau 151–372 Δ215–219 deletion mutants display very similar Kd values (∼12 µM), although with different enthalpy changes (ΔH = ‐6.6, ΔH = ‐2.7 and ΔH = ‐1.9 kcal mol^−1^, respectively), which implies different entropic contributions to the binding events. Indeed, the binding of Tau 151–243 appears mainly enthalpy‐driven (‐TΔS = ‐0.2 kcal mol^−1^), while the binding of Tau 151–372 Δ175–179 and of Tau 151–372 Δ215–219 have smaller enthalpy changes and larger entropic contributions (‐TΔS = ‐4.0 and ‐TΔS = ‐4.8 kcal mol^−1^, respectively). This difference could originate from the different length of the proteins. The Tau 151–372 Δ175–179 and Tau 151–372 Δ215–219 mutants are longer constructs, extending up to residue Glu372, and include the HSPR3 which constitutes an authentic binding site as shown by our NMR analysis (Figures 1 and 2). One possible explanation for the observed favourable entropic contribution is the burial of the numerous solvent‐exposed hydrophobic residues and prolines (as ^247^PVPMP^251^) of HSPR3 upon binding with Fyn‐SH3.

Analysis of the double mutant Tau 151–372 Δ175–179 Δ215–219 (Table 1) suggests the presence of several very‐low affinity binding sites (N_II _= 5 Kd_II _= 347 µM) that are consistent with the increase of additional binding events following the deletion of the PxxP motifs in HSPR1 and HSPR2 observed in the NMR‐binding experiment of Figure 1j. While these low‐affinity sites appear to be mainly enthalpy‐driven (‐TΔS_II_ = + 0.1 kcal mol^−1^), binding at the other site (N_I _= 1 Kd_I _= 10 µM) is characterized by a large favourable entropic contribution (‐TΔS_I_ = ‐5.0 kcal mol^−1^) that could result from the burial of hydrophobic residues of HSPR3.

We speculate that non‐canonical binding sites such as HSPR3, and additional interactions provided by the third‐fourth and seventh PxxP motifs and by non‐specific contacts with neighbouring regions could play a role in supporting the Tau‐Fyn interaction when principal PxxP motifs are turned off. A basal level of binding upon deletion of the first/second and fifth/sixth PxxP motifs was indeed observed also in our model cell line (Figure 4).

The seminal work of Ittner et al.^[^ ^4^ ^]^ in murine models demonstrated that in the absence of Tau (obtained with a knock‐out mice), postsynaptic targeting of Fyn is highly reduced, indicating that a basal level of interaction between Tau and Fyn is important to support the physiological targeting of Fyn to the post‐synapses. In these specialized neuronal areas, Fyn has the important role of phosphorylating and stabilizing NMDA receptors, thereby promoting Ca^2+^ influx and the activation of calcium‐mediated signalling pathways related to synaptic plasticity, learning and memory functions.^[^ ^36^ ^]^ Under physiological conditions the process is finely regulated since hyperactivation of the NMDARs could lead to an excessive Ca^2+^ influx resulting in excitotoxicity and neuronal death. In fact, dysfunction of NMDARs has been connected to neurodegenerative disorders, such as AD.^[^ ^36^ ^]^ The presence in Tau of multiple binding sites for the SH3 domain of Fyn would be functional in supporting Fyn targeting when single PxxP motifs are deactivated. This can occur, for example, by post‐translational modification of residues nearby or within the PxxP motifs, which could affect the interaction of Tau with SH3. A prominent modification of Tau in the brain is Ser/Thr phosphorylation, which represents an important mechanism for regulating its biological activities, such as the binding to microtubules,^[^ ^2^ ^]^ and the interaction with SH3 domains. Phospho‐mimetics obtained through single Ser/Thr‐Glu mutation at PxxP sites (such as S202E or T231E or S235E) did not show severely altered binding to Fyn‐SH3.^[^ ^21^, ^35^ ^]^ On the other hand, hyperphosphorylated Tau proteoforms, mimicked by 18 Ser/Thr‐Glu substitutions or obtained through the kinase activity of GSK3β (which phosphorylates many sites in the proline rich domain^[^ ^37^ ^]^), showed significantly reduced binding to Fyn‐SH3.^[^ ^21^ ^]^ Thus, it is possible that phosphorylation at specific sites subtly modulates Tau‐Fyn interaction and the Fyn targeting to the post synapse in response to signalling events, whereas pathological hyperphosphorylation of Tau may alter its ability to bind and target Fyn, contributing to synaptic disfunction.

## Conclusions

With this work, we propose a model of the Tau‐Fyn interaction in which Tau provides a tuneable binding interface consisting of multiple and alternative binding sites for the SH3 domain, located in the proline rich region to the beginning of the R1 repeat within the microtubule binding domain. The promiscuity of SH3 binding to multiple and non‐canonical motifs in Tau might have several functional advantages: I) deactivation of one binding site, for example through serine/threonine phosphorylation,^[^ ^21^ ^]^ can be buffered by the remaining PxxP motifs or by alternative regions; II) multiple sites in Tau, separated by long and high flexible linkers, could provide a platform for docking multiple SH3 molecules, also from different signalling factors such as scaffolds (BIN1, Grb2, p85α), phospholipases (PLCγ1, PLCγ2) and Src family of non‐receptor tyrosine protein kinases (Fyn, Src, and Lck),^[^ ^24^ ^]^ in line with the suggested role of Tau as a hub in protein‐protein interaction networks; III) on‐off interactions between different sites could allow sliding movements of SH3‐containing proteins along and outside the length of Tau proline‐rich domain.

In conclusion, our study reveals how multiple and alternative sites make the full Tau protein an adaptable sticky surface for the SH3 domain of Fyn kinase, providing a binding model potentially applicable to other SH3‐containg binding partners.

## Author Contributions

R.T., G.L., L.P., CG.B., L.M., S.C., F.M. performed and analysed experiments; L.M., S.C., M.D., M.A., F.M. supervised experimental work and interpretation; F.M. designed research and wrote the article.

## Conflict of Interests

The authors declare no conflict of interest.

## Supporting information



Supporting Information

## Data Availability

The data that support the findings of this study are available from the corresponding author upon reasonable request.
