# Supplementary material for: Multiple and Alternative Sites Make Tau Protein an Adaptable Sticky Surface for the SH3 Domain of Fyn Kinase
Source: Angew Chem Int Ed Engl. 2025 May 23;64(28):e202504292. doi: 10.1002/anie.202504292 (PMC12232886; doi:10.1002/anie.202504292)
Supplement: Supplementary file 1 — Supporting Information [file ANIE-64-e202504292-s001.pdf]

Supporting information for:

Multiple and Alternative Sites Make Tau Protein an Adaptable Sticky Surface for the SH3 Domain of Fyn Kinase

Roberto Tira [a], Giulia Leo [a], Laura Prandini [a], Carlo Giorgio Barracchia [a], Mariapina D'Onofrio [a], Luca Mollica [b], Stefano Capaldi [a], Michael Assfalg [a], Francesca Munari [a] *.

[a] Department of Biotechnology, University of Verona, Verona, Italy.

[b] Department of Medical Biotechnology and Translational Medicine, University of Milan, Milan, Italy.

**Corresponding author*

This file contains:

- Materials and methods
- Supplementary figures S1, S2, S3 and S4
- Supporting Table S1
- Supplementary references

Materials and methods

Synthetic Tau peptides 170-184, 210-224, and 241-255 were purchased from Thermofisher Scientific with no additional modification.

Isopropyl-β-D-1-tiogalattopiranoside (IPTG), Tris(2-carboxyethyl) phosphine (TECP), Ethylenediaminetetraacetic acid (EDTA), dithiothreitol (DTT), DNAse, imidazole, protease inhibitors, chromatographic resins were purchased from Merck.

$^{15}NH_4Cl$, D-glucose-^{13}C6, D_2O were purchased from Cambridge Isotope Laboratories and Merck.

Plasmids mVenus N1, mCerulean N1, and C17V were a gift from Steven Vogel (Addgene plasmid #27793 ; #27795; #26395)[1]. Plasmid pKK-FRET-ORF1-3C-Cerulean_ORF2-TEV-Venus was a gift from Andrzej Dziembowski (Addgene plasmid #105805)[2].

Cell culture media, antibiotics and FBS were purchased from Gibco or Aurogene.

Cloning, expression and purification of recombinant proteins

The Tau constructs Tau2N4R (uniprot P10636-8) and Tau151-243 were cloned, expressed and purified with a his-tag as described in[3]. Tau151-372 was cloned into a pET15 vector modified with a N-terminal his-tag cleavable with TEV protease (pET-TEV). For expression of unlabeled Tau151-372 protein, BL21(DE3) Codon Plus E. coli cells were induced at 37 °C with 0.5 mM IPTG for 5 hours in LB medium. Then, collected cells were resuspended in lysis buffer (20 mM Tris-HCl pH 7.6 containing 500 mM NaCl, 20 mM imidazole, and supplemented with protease inhibitors, DNAse and 10 mM $MgCl_2$), sonicated, and boiled for 15 minutes. After centrifugation, the supernatant was loaded into a Ni Sepharose Fast Flow (GE Healthcare), and the his-tagged protein purified by gravity flow by standard protocol. The eluted protein was then dialyzed overnight at 4°C in 20 mM Tris-HCl pH 7.6 with 150 mM NaCl, and then incubated at 25°C with TEV protease for 4 hours in the presence of 0.5 mM TCEP. The cleaved tag and the protease were removed by a further IMAC chromatography where the purified Tau151-372 was loaded with 20 mM imidazole and recovered in the flow through. Finally, the protein was dialyzed overnight at 4°C against the buffer required for the specific experiment. The mutants Tau 151-372 Δ175-179, Tau 151-372 Δ215-219 and Tau 151-372 Δ175-179 Δ215-219 were obtained by deletion mutagenesis, and the protein samples produced as for Tau151-372.

The cDNA of full-length human Fyn was retrotranscribed from the mRNA extracted from SH-SY5Y cell line. The sequence matches the current uniprot P06241-1 isoform except for the single natural variant E506. The Fyn-SH3 domain (residues 82-143) was cloned into the pET-TEV vector described before and expressed at 37 °C with 0.5 mM IPTG for 4 hours in BL21(DE3) E. coli cells. For purification and his-tag removal, we followed the same procedure described for Tau151-372, except for the boiling step which was omitted.

All constructs were verified by DNA sequencing.

Uniformly labeled ^{15}N and ^{15}N/^{13}C proteins were produced by growing bacterial cells overnight at 25°C in M9 minimal medium containing $^{15}NH_4Cl$, or $^{15}NH_4Cl$/D-glucose-$^{13}C_6$ as the sole source of isotope labeled elements.

NMR spectroscopy

The ^{1}H,^{15}N HSQC (heteronuclear single-quantum coherence) spectra of Tau2N4R, Tau151-243, Tau 151-372, and Tau151-372 deletion mutants were recorded in absence or presence of Fyn-SH3 on ^{15}N-labeled Tau samples in the concentration range of 0.1 - 0.05 mM in 20 mM potassium phosphate buffer pH 6.8, supplemented with 2.5 mM EDTA, 2 mM DTT and

8% D_2O at 278K. Spectra were acquired with the Bruker pulse program *hsqcetf3gpsi,* with 2048 (t_2, ^{1}H) × 256 (t_1, ^{15}N) complex points, spectral widths of 13.65 and 25 ppm in ^{1}H and ^{15}N dimensions, respectively, and ^{15}N carrier frequency set to 118.5 ppm (Tau2N4R) or 119 ppm (Tau151-243 and Tau151-372).

The ^{1}H,^{15}N-backbone chemical shift assignment of Tau2N4R was obtained as described in [3]. Briefly, we started from the peak list of a Tau 244-441 construct, that we previously assigned by 3D heteronuclear experiments [3], and integrated the assignment of the N-terminal part by comparing our spectra with published assignments of Tau441 (BMRB entry 50701[4]) and of shorter Tau constructs (BMRB entries: 28065[5] , 17945[6], 19253[7], and [8]). Thanks to the unfolded nature of Tau protein and of its shorter constructs, most of the peaks in the ^{1}H,^{15}N HSQC spectrum of Tau151-243 match with the corresponding peaks in the ^{1}H,^{15}N HSQC spectrum of Tau2N4R and were thus assigned by visual transfer. A few peaks belonging to residues at the N- and C- ends (151-154 and 240-243) do not match due to truncations and therefore are not assigned. Assignment of the ^{1}H,^{15}N resonances of Tau 151-372 was obtained by comparing the ^{1}H,^{15}N HSQC spectrum of Tau 151-372 with the spectra of Tau2N4R, Tau 151-243, and with the spectrum of Tau4RD (244-372) for which the assignment was already available[3]. The ^{1}H,^{15}N HSQC spectra of Tau 151-372 Δ175-179, Tau 151-372 Δ215-219 and of Tau 151-372 Δ175-179 Δ215-219 differ from the reference spectrum of Tau 151-372 only in the proximity of the deleted region. In particular, peaks belonging to the four residues before and after the deleted region do not match with the reference spectrum and were thus not included in the analysis.

The ^{1}H,^{15}N-backbone chemical shift assignment of Fyn-SH3 protein, reported in figure S2A and supporting table S1, was obtained from the analysis of 3D HNCOCACB and HNCACB experiments recorded on a 0.54 mM sample of ^{15}N,^{13}C-labeled protein in 20 mM potassium phosphate buffer pH 6.8 supplemented with 2.5 mM EDTA and 10% D_2O at 298K. Assignment of ^{1}H,^{15}N Trp side chain (sc) resonances was taken from published spectra[9,10]. The HNCACB spectrum of ^{15}N-^{13}C labeled Fyn-SH3 was recorded with the best-HNCACB Bruker pulse program *b_hncacbgp3d.2* with 698x52x90 (^{1}H x ^{15}N x ^{13}C) complex points, D1=0.2 sec, NS=8 and spectral widths of 11.9, 27 and 70 ppm in ^{1}H, ^{15}N and ^{13}C dimensions, respectively, and ^{15}N carrier frequency set to 119.5 ppm and ^{13}C carrier frequency set to 43 ppm. The HNCOCACB spectrum of ^{15}N-^{13}C labeled Fyn-SH3 was recorded with the best-HNCOCACB Bruker pulse program *b_hncocacbgp3d.2* with 698x48x80 (^{1}H x ^{15}N x ^{13}C) complex points, D1=0.2 sec, NS=8 and spectral widths of 11.9, 27 and 70 ppm in ^{1}H, ^{15}N and ^{13}C dimensions, respectively, and ^{15}N carrier frequency set to 119.5 ppm and ^{13}C carrier frequency set to 43 ppm.
The ^{1}H^{15}N HMQC (heteronuclear multiple quantum coherence) spectra of ^{15}N labeled Fyn-SH3 were recorded at 298K with the sofast-HMQC Bruker pulse program *sfhmqcf3gpph,* with 800 (t_2, ^{1}H) × 256 (t_1, ^{15}N) complex points, recycle delay D1=0.2 sec, and spectral widths of 13.65 and 27 ppm in ^{1}H and ^{15}N dimensions, respectively, and ^{15}N carrier frequency set to 119.5 ppm.

For the SH3-Tau peptides titration, samples of 0.1 mM ^{15}N-labeled Fyn-SH3 were titrated with stock solutions of unlabeled Tau peptides having the following concentrations: 2.1 mM for Tau 170-184, 1.9 mM for Tau 210-224, and 2.0 mM for Tau 241-255. Titration experiments were performed in 20 mM potassium phosphate buffer pH 6.8, 2.5 mM EDTA, and 10% D_2O, and measured by a series of sofast-HMQC spectra recorded on ^{15}N-Fyn-SH3 at 298K at every titration point.

In all NMR-binding experiments, the pH of each protein and ligand sample was carefully checked and eventually adjusted to the same value (6.8) with a micro pH electrode (Hanna), to eliminate the possible influence of pH variation on the measured NMR parameters.

The NMR spectra of ^{15}N-labeled Tau proteins and of ^{15}N-/^{15}N-^{13}C labeled Fyn-SH3 were acquired on a Bruker Avance NEO 600 MHz spectrometer equipped with a Prodigy TCI cryoprobe.
Spectra were processed using Topspin 3.6.2 (Bruker, Karlsruhe) and analyzed with the software Sparky (T. D. Goddard and D. G. Kneller, University of California, San Francisco).
The combined chemical shift perturbation (CSP) was calculated as: $CSP=[(\Delta\delta H)^2 + (\Delta\delta N/5)^2]^{0.5}$, where $\Delta\delta H$ and $\Delta\delta N$ are the chemical shift changes measured in the ^1H and ^{15}N frequency dimensions, respectively.
The NMR derived binding constants Kd of the SH3-Tau peptide interactions were obtained by nonlinear least-squares fitting the observed CSP data to the following equation: $\Delta\delta obs = \Delta\delta max\, (a + b + Kd - ((a + b + Kd)^2 - 4ab)^{1/2})/2a$, where $\Delta\delta obs$ is the measured CSP and $\Delta\delta max$ is the maximal signal change upon saturation, a and b are the total SH3 and Tau peptide concentrations, respectively.

Molecular dynamics and Docking calculation

To reconstruct the atomic-level structural ensembles of the different Tau peptides used in this work, we used Scaled Molecular Dynamics (SMD)[11] as implemented in the Biki[12] version of GROMACS[13], a technique developed for obtaining enhanced sampling of the potential energy surface and favoring the transitions between minima in a way comparable to long conventional molecular dynamics simulations. Starting structures of Tau regions 170-184 (HSPR1), 210-224 (HSPR2), and 241-255 (HSPR3) have been generated using PeptideBuilder[14] with random distribution of F/P angles. A parallelepipedal solvent box was created around the peptides, solvated with 9250 TIP3P[15] water molecules. The overall system charge was balanced with counterions. After minimization with the steepest descent method (with a convergence of the total force equal to 100 kJ mol^{-1} nm^{-1}), the system was equilibrated (with isotropic positional restraints on protein heavy atoms, k = 200 kJ mol^{-1} nm^{-2}) for 500 ps in the NPT ensemble with p = 1 atm and T = 300 K, then for 500 ps in the NVT ensemble at T = 300 K, then we performed for each system a 200 ns scaled molecular dynamics simulation in the NVT ensemble employing a timestep of 2 fs and constraining all covalent bond lengths with the LINCS[16] algorithm and an SMD scaling factor λ= 0.75. A single linkage cluster analysis of the trajectories led to the extraction of representative structures for HSPR1, HSPR2 and HSPR3 peptides (respectively: 7, 11, 7) further used for docking calculations with the Fyn SH3 domain.

All the docking calculations reported in the article have been performed using the HADDOCK[17] software, a collection of Python and CNS[18] (Crystallography and NMR System) scripts that allow data-driven docking calculations. Data driven docking has been performed using the chemical shifts perturbation (CSP) of the SH3 domain reported for each titration due to their statistical relevance (i.e., CSP greater than the average plus standard deviation) and has been converted in ambiguous restraints for docking calculations.
Inter- and intramolecular energies are evaluated using full electrostatic and van der Waals energy terms with an 8.5 Å cut-off with a shifting function for the electrostatic energy and switching function between 6.5 and 8.5 Å for the van der Waals energy using the OPLS[19] nonbonded parameters. The docking protocol used in the present work consists of randomization of orientations and rigid body energy minimization of the complexes formed by HSPR1, HSPR2 and HSPR3 peptides (cyclically used as an ensemble of ligands for each system) and Fyn-SH3 domain structure (1shf PDB file[20]), considering their spatial organization not influenced by Tau binding according to experimental data. In the randomization stage, the two molecular partners are placed at 150 Å from each other in space and each molecule is randomly rotated

around its center of mass. Rigid body energy minimization is then performed, i.e., each macromolecule involved in the binding is kept rigid, with four cycles of orientational optimization in which each protein in turn is allowed to rotate to minimize the intermolecular energy function. Then, both translations and rotations are allowed, and the molecules are docked by rigid body energy minimization. During these steps the solvent has been treated implicitly using a dielectric constant of 10, considered the best value for representing the internal electrostatic behavior of protein complexes. For each complex 1000 structures have been generated and analyzed as reported in the main text in terms of internal contacts and binding energy.

Isothermal Titration Calorimetry (ITC)

For ITC measurements, purified Fyn-SH3 and Tau proteins were dialyzed overnight against 20 mM potassium phosphate buffer (pH 6.8). Concentrations of proteins were measured using a NanoDrop instrument (Thermo Fisher Scientific), by using molar extinction coefficients at 280 nm. Calorimetric titrations were carried out on a MicroCal PEAQ-ITC instrument (Malvern) at 25 °C. The sample cell contained 20 µM Tau protein and the injection syringe 0.6 mM or 1 mM solution of Fyn-SH3. Only for the Tau mutant 151-372 Δ175-179 Δ215-219, the experiment was conducted with 60 µM Tau protein in the cell and 1.2 mM SH3 in the syringe. Titrations were carried out with a preliminary 0.4 µl injection, followed by 2 µl injections with time intervals of 150 sec, with stirring rate of 500 rpm and reference power of 10 µcal/sec. A blank titration (ligand into buffer) was recorded in the same experimental conditions and the dilution heats were subtracted to the data. Binding isotherms were analyzed with the MicroCal PEAQ-ITC analysis software, using the two-sets of sites or the one-set of sites fitting models.

Plasmids construction for FRET measurements

The pKK-FRET vector for the co-expression of mCerulean/mVenus-tagged proteins in mammalian cells and the FRET standard vector C17V were obtained from Addgene (cat. n. 105805 and 26395). The cDNA sequences of human FYN 1-537 and of Tau2N4R (or its mutants) were amplified by PCR and cloned into the NotI/MluI and BamHI/EcoRV restriction sites of the pKK-FRET vector, respectively. The resulting constructs allow to express the target proteins fused at their C-terminus to mCerulean (FYN) and mVenus (Tau) FRET pair. The empty pKK vector, expressing unfused donor (mCerulean) and acceptor (mVenus), and a construct expressing FYN-mCerulean and free mVenus were used as negative FRET controls. All constructs were verified by DNA sequencing.

Cell culture and transfection

Hek293T cells were cultured in T-flasks using Dulbecco's Modified Eagle's Medium (DMEM) supplemented with 10% (v/v) Fetal Bovine Serum (FBS), 1% penicillin/streptomycin and 1% glutamine at 37 °C in a humidified atmosphere with 5% CO_2. For FRET experiments, cells were seeded at a density of 50000 cells/well on glass coverslips and left to adhere overnight. The next day, cells were transfected with 500 ng of plasmid DNA using 0.5% Lipofectamine LTX (Invitrogen). After 24 hours, the cells were washed three times with PBS and fixed with ice-cold 4% PFA for 20 minutes at room temperature. After three additional washes with PBS, the coverslips were mounted on glass slides with fluorescence mounting medium (Dako) and sealed with nail polish.

Laser scanning confocal microscopy

All images were acquired with a confocal laser scanning microscope Evident FV4000 using a 60X oil immersion objective with pixel size of 90 nm. The donor and transfer channels were excited at 445 nm and detected between 457-597 nm and 541-621 nm, respectively. In the acceptor channel the excitation was set to 514 nm and the detection interval was 547-627 nm. The acquisition parameters (laser power, detector gain and pixel size) were kept constant for all the imaged samples.

Image analysis

Image processing and analysis were carried out with Fiji[21]. The spectral bleed-through (SBT) of the donor and acceptor was determined using samples of cells transfected with plasmid expressing only free mCerulean (mCerulean-N1, Addgene cat. n. 27795) and mVenus (mVenus-N1, Addgene cat. n. 27793), respectively, after background subtraction. Raw and normalized FRET (NFRET) values were calculated with the plugin PixFRET[22] with a threshold of ten times the background intensity and a Gaussian blur factor of 2. For statistical analysis, the mean FRET intensity, normalized for donor and acceptor expression levels (NFERT)[23], of multiple individual cells was measured in at least five different images taken from two biological replicates for each pair.

Statistical significance was assessed by one-way ANOVA followed by Tukey's HSD post hoc test for pairwise comparisons. The method was validated using a plasmid expressing the FRET-positive C17V standard, in which mCerulean is fused with mVenus via a 17-residue linker, and the empty vector pKK-FRET expressing the two separate fluorescent proteins (supplementary figure S4).

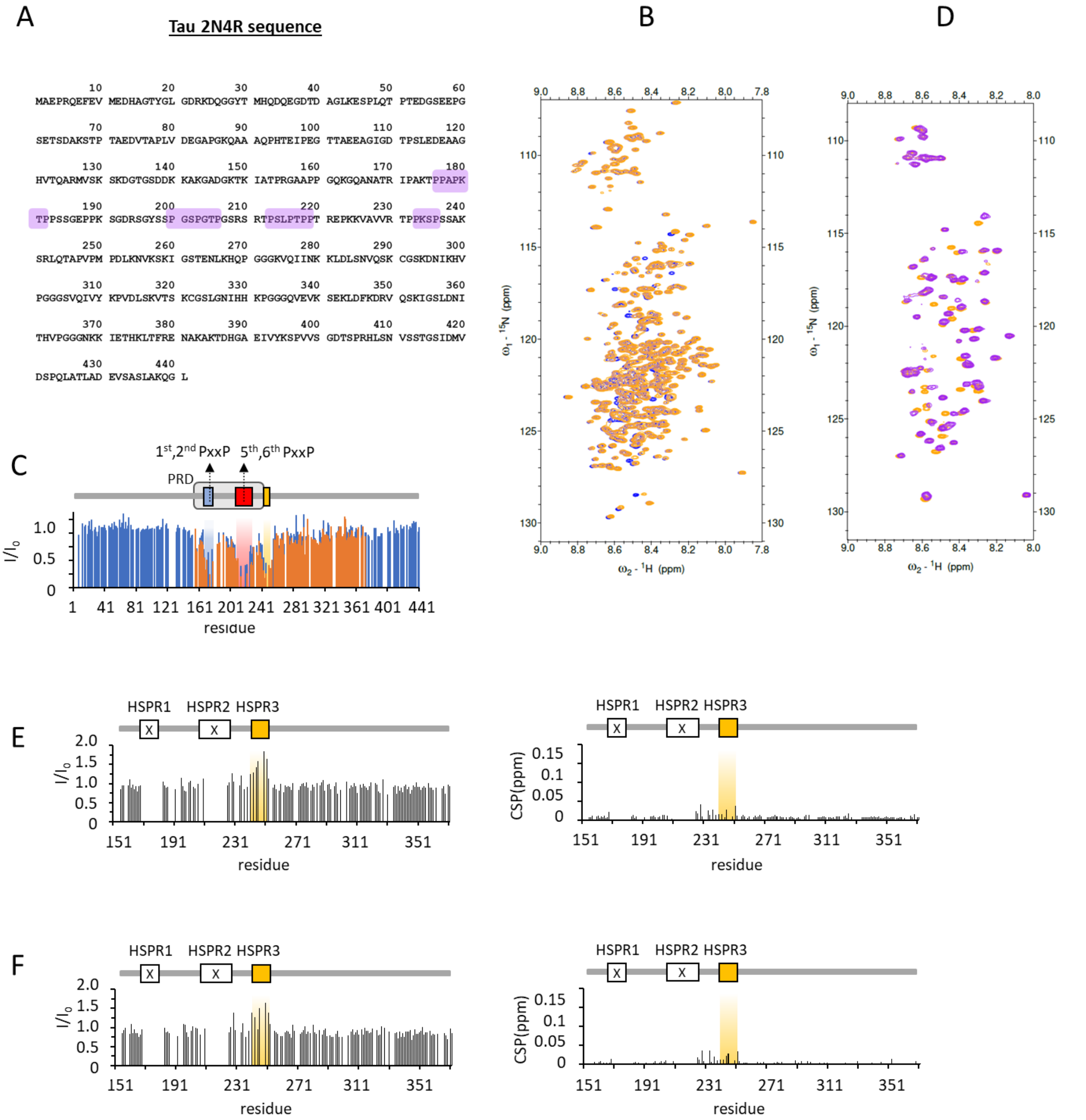

Figure S1: A) Amino acids sequence of Tau (isoform 2N4R) where the 7 PxxP motifs (1st-7th, partially overlapped) are highlighted in purple. B) $^{1}H,^{15}N$-HSQC spectra overlay of ^{15}N-Tau 2N4R alone (blue) and in presence of Fyn-SH3 at 1:1 molar ratio (yellow). C) Superimposition of the NMR binding profiles of Tau2N4R (blue bars, data from figure 1D) and of Tau151-372 (orange bars, data from figure 1F) to directly compare the pattern of reduction in signals intensity of the two Tau constructs upon binding to Fyn-SH3. The scheme of the main regions affected (highlighted with colored boxes), is shown on the top. D) Overlay of $^{1}H,^{15}N$-HSQC spectra of Tau151-243 in the free form (yellow) and in the presence of Fyn-SH3 at 1:1 molar ratio (purple). E-F) NMR-based competition experiments. Intensity ratio (I/I_0) of NMR signals from $^{1}H,^{15}N$ spectra of ^{15}N-Tau 151-372 Δ175-179 Δ215-219 acquired in presence of Fyn-SH3 at 1:1 molar ratio (I_0), or in presence of Fyn-SH3 and Tau 170-184 (E) or Tau 210-224 (F) at 1:1:5 molar ratio (I), versus the Tau sequence. The CSP plots are shown on the right. Residues affected by signal overlap were excluded from the analysis. The scheme of the deletions (x) and of the main regions affected (highlighted with colored boxes), is shown on the top.

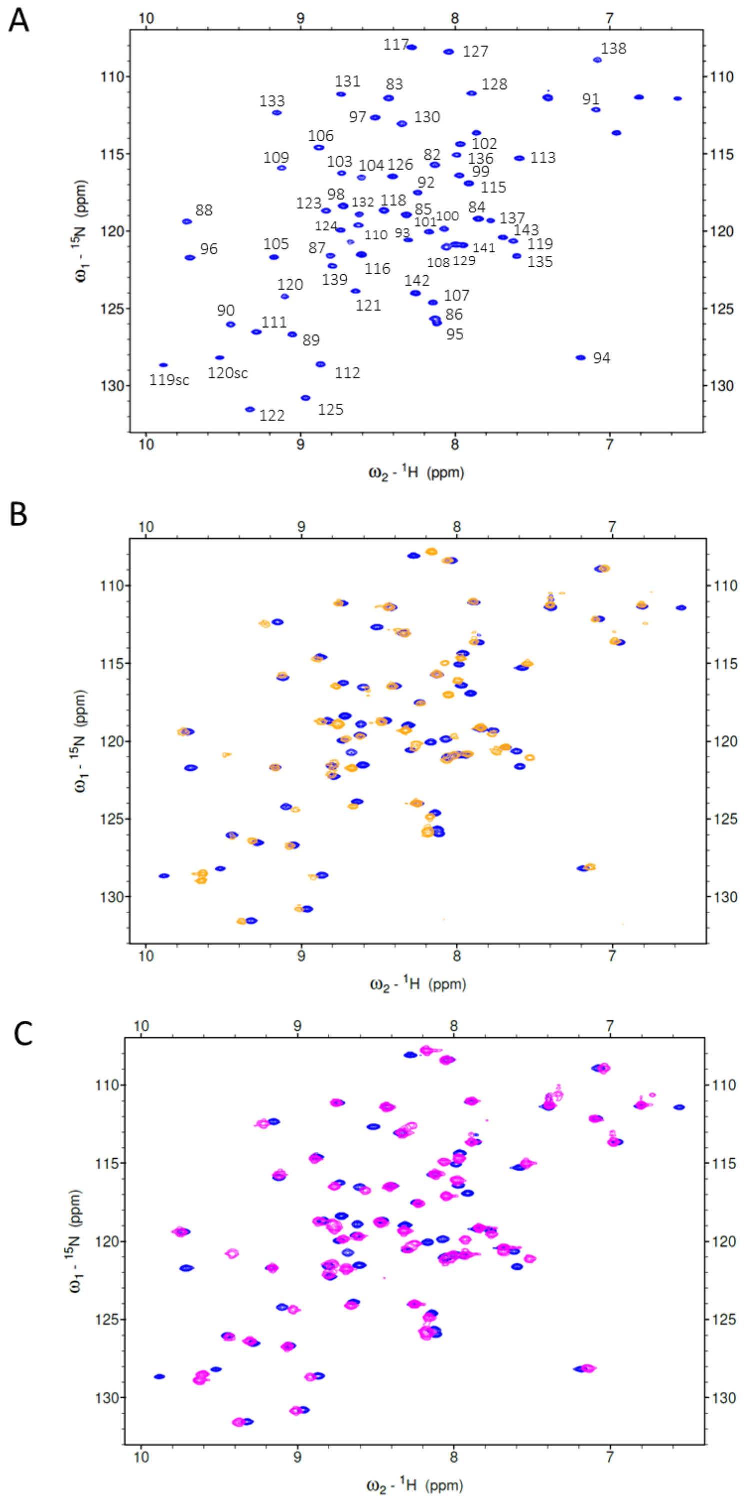

Figure S2: A) $^{1}H,^{15}N$-HMQC spectrum of Fyn-SH3 with assigned resonances; B) overlay of $^{1}H,^{15}N$-HMQC spectra of Fyn-SH3 in the free form (blue) and in the presence of Tau 151-372 at 1:1 molar ratio (yellow); C) overlay of $^{1}H,^{15}N$-HMQC spectra of Fyn-SH3 in the free form (blue) and in the presence of Tau 151-243 at 1:1 molar ratio (magenta).

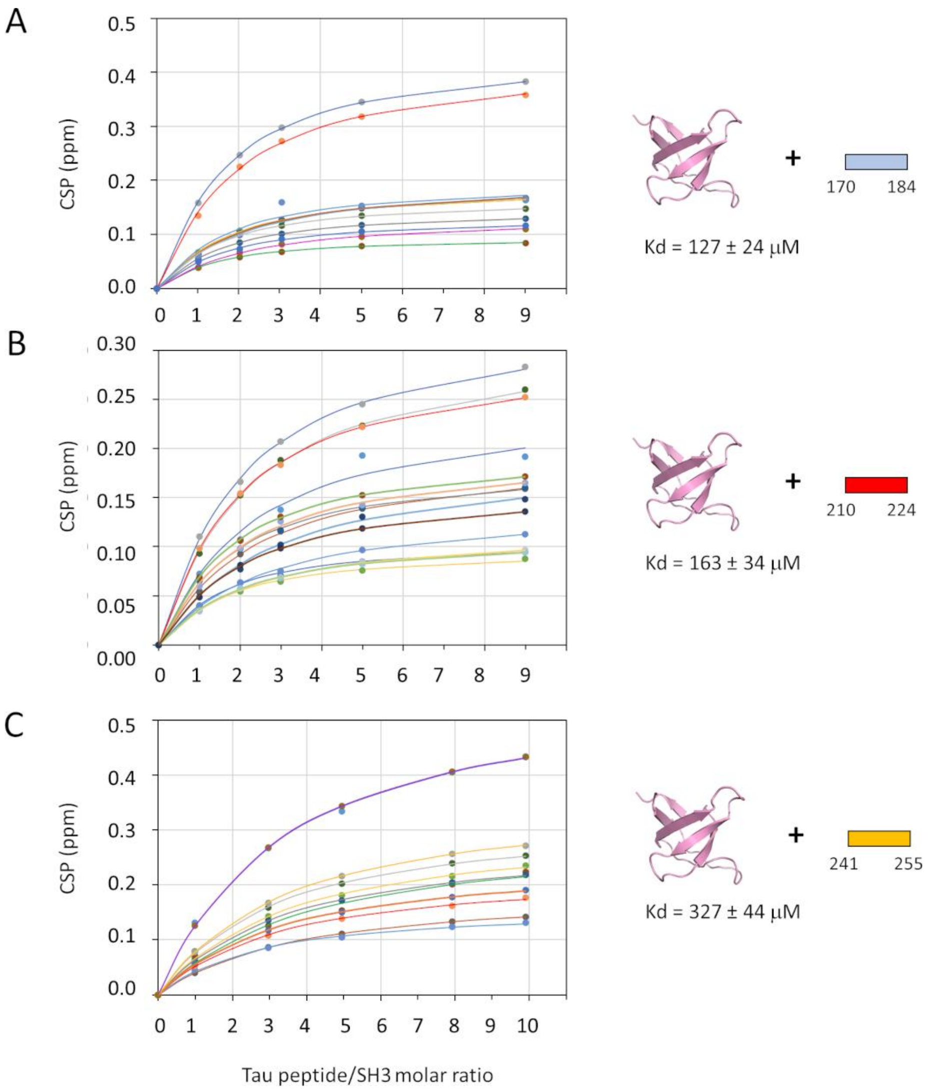

Figure S3: Binding isotherms based on CSP data from titration experiments of ^{15}N-Fyn-SH3 with Tau peptides 170-184 (A), 210-224 (B), and 241-255 (C). Resonances in fast-exchange regime from spectra reported in figures 2F, 2G and 2H were analyzed. Dots represent experimental values, while lines represent the nonlinear least-squares fitting based on a single-site binding model. Reported Kd values and corresponding standard deviation were determined based on the average of Kd values obtained from the analysis of 12 (A), 15 (B), and 12 (C) binding isotherms.

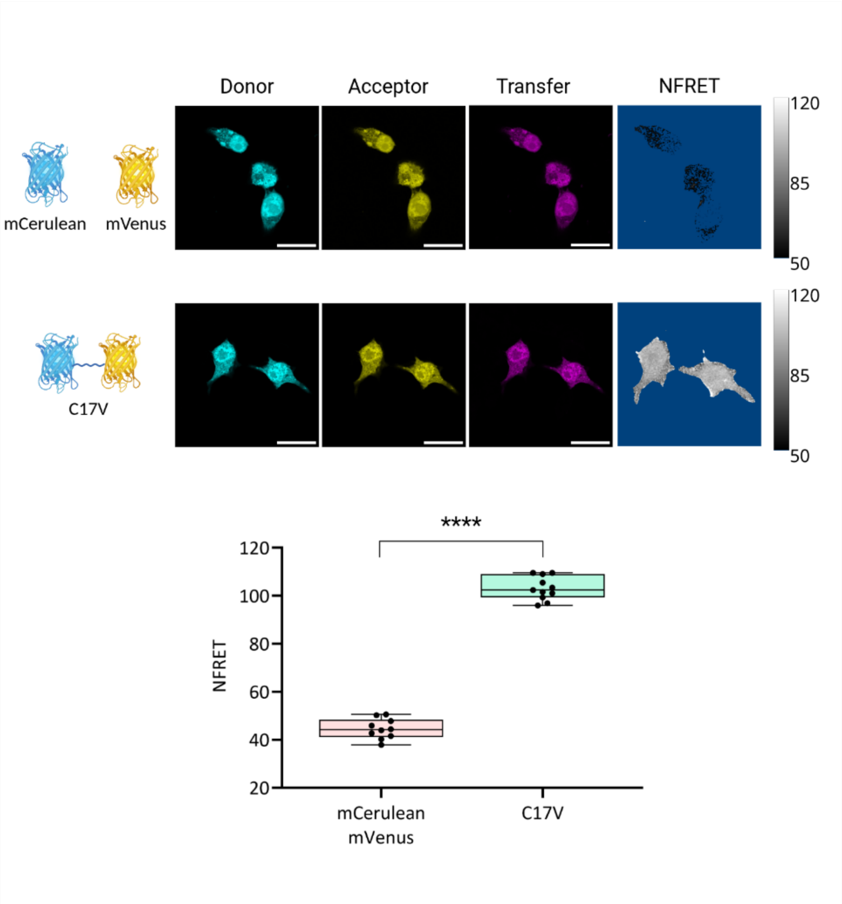

Figure S4: Representative images of the three fluorescence channels (donor, acceptor and transfer channel) and the normalized FRET (NFRET) for isolated, not fused mCerulean/mVenus (top) and the FRET positive construct C17V (bottom). The scale bars are 25 μm. NFRET images are shown with the same greyscale level. Box-plots showing the distribution of mean NFRET intensity in single cells are reported below. The experimental values are shown as black dots (**** p<0.0005, N = 10 and 11).

Table S1: ^{1}H,^{15}N-backbone chemical shift assignment of human Fyn-SH3.

	^{15}N (ppm)	^{1}H (ppm)
T82	115.626	8.130
G83	111.332	8.434
V84	119.131	7.852
T85	118.985	8.324
L86	125.633	8.146
F87	121.503	8.809
V88	119.336	9.744
A89	126.626	9.062
L90	125.965	9.458
Y91	112.092	7.097
D92	117.455	8.245
Y93	120.528	8.313
E94	128.142	7.191
A95	125.882	8.122
R96	121.642	9.714
T97	112.573	8.513
E98	118.345	8.728
D99	116.359	7.978
D100	119.832	8.070
L101	120.042	8.188
S102	114.311	7.965
F103	116.217	8.741
H104	116.539	8.614
K105	121.618	9.176
G106	114.535	8.888
E107	124.560	8.152
K108	120.995	8.057
F109	115.858	9.132
Q110	119.558	8.632
I111	126.451	9.291
L112	128.565	8.879
N113	115.197	7.579
S114	120.724	8.696
S115	116.885	7.927
E116	121.475	8.619
G117	107.993	8.283
D118	118.633	8.466
W119	120.591	7.617
W120	124.174	9.110
E121	123.824	8.651
A122	131.479	9.339
R123	118.631	8.846
S124	119.898	8.744
L125	130.740	8.976

T126	116.379	8.409
T127	108.321	8.044
G128	111.014	7.902
E129	120.825	8.002
T130	112.965	8.346
G131	111.051	8.746
Y132	118.852	8.631
I133	112.280	9.165
S135	121.541	7.602
N136	114.986	7.994
Y137	119.265	7.772
V138	108.861	7.086
A139	122.196	8.799
V141	120.834	7.955
D142	123.955	8.261
S143	120.362	7.694

Supplementary references

[1] S. V. Koushik, H. Chen, C. Thaler, H. L. Puhl, S. S. Vogel, *Biophys. J.* **2006**, *91*, L99–L101.

[2] R. J. Szczesny, K. Kowalska, K. Klosowska-Kosicka, A. Chlebowski, E. P. Owczarek, Z. Warkocki, T. M. Kulinski, D. Adamska, K. Affek, A. Jedroszkowiak, A. V. Kotrys, R. Tomecki, P. S. Krawczyk, L. S. Borowski, A. Dziembowski, *PloS One* **2018**, *13*, e0194887.

[3] F. Munari, L. Mollica, C. Valente, F. Parolini, E. A. Kachoie, G. Arrigoni, M. D'Onofrio, S. Capaldi, M. Assfalg, *Angew. Chem. Int. Ed.* **2022**, *61*, DOI 10.1002/anie.202112374.

[4] R. L. Narayanan, U. H. N. Dürr, S. Bibow, J. Biernat, E. Mandelkow, M. Zweckstetter, *J. Am. Chem. Soc.* **2010**, *132*, 11906–11907.

[5] T. Ukmar-Godec, P. Fang, A. Ibáñez De Opakua, F. Henneberg, A. Godec, K.-T. Pan, M.-S. Cima-Omori, A. Chari, E. Mandelkow, H. Urlaub, M. Zweckstetter, *Sci. Adv.* **2020**, *6*, eaba3916.

[6] N. Sibille, I. Huvent, C. Fauquant, D. Verdegem, L. Amniai, A. Leroy, J. Wieruszeski, G. Lippens, I. Landrieu, *Proteins Struct. Funct. Bioinforma.* **2012**, *80*, 454–462.

[7] P. Barré, D. Eliezer, *Protein Sci. Publ. Protein Soc.* **2013**, *22*, 1037–1048.

[8] N. W. Harbison, S. Bhattacharya, D. Eliezer, *PLoS ONE* **2012**, *7*, e34679.

[9] T. K. Mal, S. J. Matthews, H. Kovacs, I. D. Campbell, J. Boyd, *J. Biomol. NMR* **1998**, *12*, 259–276.

[10] K.-S. Pi, D. Bortolotti, Y. Sang, G. Schiuma, S. Beltrami, S. Rizzo, A. Bortoluzzi, E. Baldi, A. L. Creagh, C. A. Haynes, R. Rizzo, S. K. Straus, *Viruses* **2022**, *14*, 2384.

[11] W. Sinko, Y. Miao, C. A. F. De Oliveira, J. A. McCammon, *J. Phys. Chem. B* **2013**, *117*, 12759–12768.

[12] S. Decherchi, G. Bottegoni, A. Spitaleri, W. Rocchia, A. Cavalli, *J. Chem. Inf. Model.* **2018**, *58*, 219–224.

[13] M. J. Abraham, T. Murtola, R. Schulz, S. Páll, J. C. Smith, B. Hess, E. Lindahl, *SoftwareX* **2015**, *1–2*, 19–25.

[14] M. Z. Tien, D. K. Sydykova, A. G. Meyer, C. O. Wilke, *PeerJ* **2013**, *1*, e80.

[15] W. L. Jorgensen, J. Chandrasekhar, J. D. Madura, R. W. Impey, M. L. Klein, *J. Chem. Phys.* **1983**, *79*, 926–935.

[16] B. Hess, H. Bekker, H. J. C. Berendsen, J. G. E. M. Fraaije, *J. Comput. Chem.* **1997**, *18*, 1463–1472.

[17] C. Dominguez, R. Boelens, A. M. J. J. Bonvin, *J. Am. Chem. Soc.* **2003**, *125*, 1731–1737.

[18] A. T. Brünger, P. D. Adams, G. M. Clore, W. L. DeLano, P. Gros, R. W. Grosse-Kunstleve, J. S. Jiang, J. Kuszewski, M. Nilges, N. S. Pannu, R. J. Read, L. M. Rice, T. Simonson, G. L. Warren, *Acta Crystallogr. D Biol. Crystallogr.* **1998**, *54*, 905–921.

[19] W. L. Jorgensen, D. S. Maxwell, J. Tirado-Rives, *J. Am. Chem. Soc.* **1996**, *118*, 11225–11236.

[20] M. E. Noble, A. Musacchio, M. Saraste, S. A. Courtneidge, R. K. Wierenga, *EMBO J.* **1993**, *12*, 2617–2624.

[21] J. Schindelin, I. Arganda-Carreras, E. Frise, V. Kaynig, M. Longair, T. Pietzsch, S. Preibisch, C. Rueden, S. Saalfeld, B. Schmid, J.-Y. Tinevez, D. J. White, V. Hartenstein, K. Eliceiri, P. Tomancak, A. Cardona, *Nat. Methods* **2012**, *9*, 676–682.

[22] J. N. Feige, D. Sage, W. Wahli, B. Desvergne, L. Gelman, *Microsc. Res. Tech.* **2005**, *68*, 51–58.

[23] Z. Xia, Y. Liu, *Biophys. J.* **2001**, *81*, 2395–2402.
